# Development of an alarm symptom-based risk prediction score for localized oesophagogastric adenocarcinoma (VIOLA score)☆

**DOI:** 10.1016/j.esmoop.2022.100519

**Published:** 2022-06-24

**Authors:** H.C. Puhr, R. Puhr, D.A. Kuchling, L. Jahic, J. Takats, T.J. Reiter, M. Paireder, G. Jomrich, S.F. Schoppmann, A.S. Berghoff, M. Preusser, A. Ilhan-Mutlu

**Affiliations:** 1 Department of Medicine I, Division of Oncology, Medical University of Vienna, Vienna, Austria; 2 Center for Medical Statistics, Informatics and Intelligent Systems, Medical University of Vienna, Vienna, Austria; 3 Department of Surgery, Medical University of Vienna, Vienna, Austria

**Keywords:** gastric cancer, oesophageal cancer, prognosis, survival, symptoms

## Abstract

**Background:**

Gastroesophageal adenocarcinoma is a major contributor to global disease burden with poor prognosis even in resectable, regionally limited stages. Feasible prognostic tools are crucial to improve patient management, yet scarce.

**Patients and methods:**

Disease-related symptoms, patient, tumour, treatment as well as laboratory parameters at initial diagnosis and overall survival (OS) of patients with stage II and III gastroesophageal adenocarcinoma, who were treated between 1990 and 2020 at the Medical University of Vienna, were evaluated in a cross-validation model to develop a feasible risk prediction score.

**Results:**

In total, 628 patients were included in this single-centre analysis. The final score ranked from 0 to 10 and included the factors sex (female +1), age, years (30-59 +1, >60 +2), underweight classified by body mass index (+2), location of the tumour (stomach +1), stage (III +2), stenosis in endoscopy (+1) and weight loss (+1). The score was grouped into low- (0-3), medium- (4-6) and high-risk (7+) subgroups. The median OS were 70.3 [95% confidence interval (CI) 51.2-111.8], 23.4 (95% CI 21.2-26.7) and 12.6 (7.0-16.1) months, respectively. The 1-year survival probabilities were 0.88 (95% CI 0.83-0.93), 0.75 (95% CI 0.70-0.79) and 0.54 (95% CI 0.39-0.74), whereas the 5-year survival probabilities were 0.57 (95% CI 0.49-0.66), 0.24 (95% CI 0.20-0.28) and 0.09 (95% CI 0.03-0.28), respectively.

**Conclusions:**

The VIennese risk prediction score for Oesophagogastric Localized Adenocarcinoma (VIOLA) risk prediction score poses a feasible tool for the estimation of OS in patients with regionally limited gastroesophageal adenocarcinoma and, thus, may improve patient management in clinical routine. Prospective analyses should be carried out to confirm our findings.

## Highlights

• Feasible prognostic tools for resectable gastroesophageal cancer are highly needed.
• Alarm symptoms, patient and tumour characteristics are associated with prognosis.
• The VIOLA risk prediction score is based on these three prognostic categories.
• Sex, age, body mass index, location of the tumour, stage, stenosis and weight loss are included.
• It is a feasible tool to help clinicians and patients with treatment decisions.

## Introduction

Gastroesophageal cancer is a devastating disease and a major contributor to global disease burden.^1^^,^^2^ Although cancer of the upper gastrointestinal tract is more frequent in Asian countries, there has been a rapid increase of adenocarcinomas in Western populations in recent years.^3^^,^^4^ It is surmised that tumour biology and, thus, response to treatment and overall survival (OS) show variations between ethnicities.^5^^,^^6^ Yet, prognosis remains poor independent of ethnical background^7^ and even in regionally limited, resectable stages.^8^^,^^9^ Thus new prognostic tools to improve patient management are crucial, yet scarce. In particular, patients with stage II or III adenocarcinoma, who are prone to disease recurrence, might profit from a feasible prognostic score.^10^^,^^11^

As there currently are no screening methods for gastroesophageal cancer implemented in European patient care, a large part of diagnoses is made after patients experience distinguishing symptoms and seek medical advice.^12^ Although there is no standard definition, the so-called alarm symptoms include dysphagia, dyspepsia, weight loss and gastroesophageal bleeding with iron-deficiency anemia,^13^^,^^14^ and are surmised to be associated with the OS.^15^

Other prognostic factors are patient characteristics such as sex and age, as well as tumour characteristics such as location of the tumour (stomach, gastroesophageal junction, oesophagus) and tumour stage.^8^^,^^9^^,^^16^ Furthermore, laboratory results that are associated with organ function such as total blood count, bilirubin and creatinine, nutrition such as serum albumin, systemic inflammation such as C-reactive protein (CRP) as well as tumour markers such as carcinoembryonic antigen (CEA) and carbohydrate antigen 19-9 (CA19-9) are surmised to have prognostic value in patients with gastroesophageal cancer.^17^, ^18^, ^19^, ^20^, ^21^ As alarm symptoms, patient and tumour characteristics as well as laboratory results are easily retrievable parameters at first diagnosis, a prognostic score based on these markers might provide a feasible tool to estimate outcome and improve patient management early on. Thus the aim of this retrospective single-centre analysis was to create a feasible prognostic score in a large European cohort, which can easily be implemented in clinical routine to help clinicians as well as patients with treatment decisions.

## Methods

### Data collection

For this single-centre analysis we collected data from patients who fulfilled the following criteria: age ≥18 years; histologically proven localized (clinical stage II and III evaluated for this analysis by International Union Against Cancer (Union Internationale Contre le Cancer) (UICC) TNM (tumour–node–metastasis) Classification of Malignant Tumours—8th edition) gastroesophageal adenocarcinoma; and cancer treatment at the Medical University of Vienna between January 1990 and December 2020.

Patients who were not treated at the Medical University of Vienna (i.e. patients who consulted the hospital for a second opinion only) were not included in this analysis. Eligible patients were identified from the hospital-owned patient database of the General Hospital Vienna, Medical University Vienna, Austria. Tumour staging prior to therapy was mandatory and carried out according to the hospital standard. The treatment decision was made according to the individual decision of an interdisciplinary tumour board, which ensured the best possible treatment according to the respective standard of knowledge at the time of diagnosis. The treatment included systemic chemotherapy and/or gastrectomy and/or radiation therapy of the primary tumour.

Clinical information including patient demographics, symptoms, laboratory parameters and survival outcome was obtained and stored in a password-secured FileMaker Pro-based database located on the servers of the Division of Oncology at the Medical University of Vienna. The following parameters were assessed as alarm symptoms at the time of first diagnosis: dysphagia, dyspepsia, weight loss, stenosis in the endoscopy, active bleeding and ulcers in the endoscopy, frailty. Dysphagia was classified as positive (=dysphagia ‘yes’) when moderate (able to eat some solid foods) or severe dysphagia (able to swallow liquids only) was present at the time of first diagnosis. No further grading of this symptom was applied within the scope of this analysis. When involuntary weight loss at first diagnosis was documented in the patient’s history, it was classified as positive without further grading. Frailty at first diagnosis was assessed either by Eastern Cooperative Oncology Group performance status, which is routinely recorded in the patient’s history before starting therapy and was classified as ‘frail’ when ≥2, or by some more detailed patient information recorded when first diagnosed (i.e. too frail for resection, the patient received best supportive care).

In addition to alarm symptoms, patient characteristics [gender, age at first diagnosis, second oncology before or at the same time as gastroesophageal cancer diagnosis, family history, year of cancer diagnosis, body mass index (BMI)] as well as routinely assessed tumour characteristics [location, stage, human epidermal growth factor receptor 2 (HER2) positivity], treatment characteristics (chemotherapy at initial diagnosis, surgical resection, radiation therapy) and laboratory parameters at first diagnosis [organ function (total blood count, bilirubin, creatinine), infection (CRP), nutrition (albumin), tumour markers (CEA and CA19-9)] were evaluated.

Laboratory parameters were categorized in ‘within/above/below the normal limit’ according to clinical management (reference levels: haemoglobin ≥12.0 g/dl, platelets 150-350 G/l, white blood cells 4.0-10.0 G/l, CRP ≤0.5 mg/dl, albumin ≥ 35 g/l, creatinine ≤1.2 mg/dl, bilirubin ≤1.2 mg/dl, CEA ≤5.5 μg/l, CA19-9 ≤27 kU/l). The reference levels for BMI were classified as 18.5-24.9 kg/m^2^ normal weight, ≥25.0 kg/m^2^ overweight, <18.5 kg/m^2^ underweight.

### Statistical analysis

We calculated OS as the time from the date of a patient’s first diagnosis to either date of death or date of last follow-up visit. Patients without an event were censored at the final recorded clinical visit. Univariate comparisons of OS were carried out by Kaplan–Meier survival estimates and log-rank tests as well as Cox proportional hazard models. All variables but surgery were known at baseline (including the decision for initial systemic therapy) and hence entered the models as baseline variables. Surgery was treated as a time-updated variable with a starting value of 0 at baseline, switching to 1 on the day of surgery.

Variable selection for the multivariable Cox proportional hazard model underlying the prognostic score was carried out by a fivefold cross-validation approach. In each set, stepwise variable selection by Akaike’s information criterion starting from the full model was carried out. Goodness of fit was assessed by comparing Harrell’s concordance statistic of the training and validation sets in each fold. Variables that were included in at least four of five resulting models were considered for the final model. In a last step further model reduction through stepwise variable selection by Akaike’s information criterion was allowed. The proportional hazard assumption in the final model was assessed with Schoenfeld residuals.

Statistical tests were two sided and *P* values <0.05 were considered statistically significant. All analyses were conducted using R (version 4.1.1; R Foundation for Statistical Computing, Vienna, Austria) with packages survival (V3.2-11), caret (V6.0-90) and MASS (V7.3-54) and SPSS (Version 27; IBM, New York, NY).

### Ethical considerations

All procedures followed were in accordance with the Helsinki Declaration of 1964 and later versions. Because of its retrospective design, no separate informed consent was necessary within the scope of this study.

Statistical analysis was carried out in a pseudonymized form, with a patient ID automatically created by the FileMaker Pro database application. All patient data were acquired and stored according to the current data protection law of the European Union. This analysis was approved by the ethics committee of the Medical University of Vienna (identification number 1600/2021).

## Results

### Patient and tumour characteristics

We included 628 patients with resectable gastroesophageal cancer in this analysis. At the time of data cut-off (14 August 2021), 484 (77.1%) patients were already deceased. Baseline demographic and treatment characteristics and their association with the OS in univariate analyses are shown in Supplementary Table S1, available at https://doi.org/10.1016/j.esmoop.2022.100519. Sex (*P* = 0.005), age (*P* = 0.002), BMI (*P* = 0.010), stage (*P* <0.001) and surgery (*P* <0.001) were associated with the OS.

### Laboratory parameters

Several patients had laboratory parameters above or below the normal limit (Supplementary Table S2, available at https://doi.org/10.1016/j.esmoop.2022.100519). However, median levels of laboratory parameters of the overall cohort were within the normal limit: haemoglobin, 13.3 mg/dl [standard deviation (SD) 2.47]; platelets, 259 G/l (SD 99); white blood cells, 7.18 G/l (SD 2.43); CRP, 0.5 mg/dl (SD 3.57); albumin, 41.2 g/l (SD 5.3); creatinine, 0.95 md/dl (SD 0.24); bilirubin, 0.58 mg/dl (SD 0.41); CEA, 2.5 μg/l (SD 42.1); CA19-9, 14.2 kU/l (SD 370.5). Alterations of CRP (*P* = 0.018), albumin (*P* = 0.021), CEA (*P* = 0.023) and CA19-9 (*P* = 0.002) were associated with the OS in univariate analyses.

### Symptoms

Dysphagia (50.48% of patients) and dyspepsia (59.55%) were the most common symptoms, followed by weight loss (43.95%) and stenosis in endoscopy (31.37%). All these symptoms were associated with the OS in univariate analyses, whereas less common symptoms such as weakness (10.03%) and active gastrointestinal bleeding were not (7.96%). More detailed results are shown in Supplementary Table S3, available at https://doi.org/10.1016/j.esmoop.2022.100519.

### Development of the VIOLA score (VIennese risk prediction score for Oesophagogastric Localized Adenocarcinoma)

To ascertain the association of parameters with the OS, further evaluation was carried out with a cross-validation approach. All predefined demographic and treatment, laboratory and symptom variables (see Methods section), which were recorded at the time of the diagnosis, were included in the model building process. The individual variable selection results of the five folds in the cross-validation and the final model are presented in Supplementary Tables S4 and S5, available at https://doi.org/10.1016/j.esmoop.2022.100519, respectively.

Regarding the score distribution in our cohort, 8 (1.3%) patients had 1, 32 (5.1%) patients had 2, 119 (18.9%) patients had 3, 163 (26%) patients had 4, 165 (26.3%) patients had 5, 108 (17.2%) patients had 6 and 33 (5.3%) patients had 7 points. No participants scored >7 points. Based on the OS by score (see Supplementary Figure S1, available at https://doi.org/10.1016/j.esmoop.2022.100519 and Supplementary Table S6, available at https://doi.org/10.1016/j.esmoop.2022.100519), the score could be further grouped into low-(0-3), medium- (4-6) and high-risk (7+) subgroups. A total of 159 (25.3%) patients were categorized in the low-, 436 (69.4%) in the medium- and 33 (5.3%) in the high-risk group. The median OS was 70.3 months [95% confidence interval (CI) 51.2-111.8] in the low-, 23.4 months (95% CI 21.2-26.7 months) in the medium- and 12.6 months (7.0-16.1 months) in the high-risk group. Table 1 shows the variables by score categories.

**Table 1:** Variables by score category (percentages by column)

	Low risk (0-3)	Medium risk (4-6)	High risk (7+)
**Sex, *n* (%)**			
Male	131 (82.4)	308 (70.6)	7 (21.2)
Female	28 (17.6)	128 (29.4)	26 (78.8)
**Age (years)**			
Mean	58.7	65.8	72.1
SD	11.9	11.3	8.7
**BMI (kg/m**^**2**^**), *n* (%)**			
Normal	34 (21.4)	155 (345.6)	13 (39.4)
Above normal	57 (35.8)	138 (31.7)	10 (30.3)
Below normal	4 (2.5)	12 (2.8)	2 (6.1)
NA	64 (40.3)	131 (30)	8 (24.2)
**Location of primary tumour), *n* (%)**			
Gastroesophageal junction	69 (43.4)	199 (45.6)	7 (21.2)
Stomach	74 (46.5)	180 (41.3)	26 (78.8)
Oesophagus	16 (10.1)	57 (13.1)	0 (0)
**Stage, *n* (%)**			
Stage 2	133 (83.6)	96 (22)	0 (0)
Stage 3	26 (16.4)	340 (78)	33 (100)
**Stenosis in endoscopy, *n* (%)**			
No	97 (61)	190 (43.6)	12 (36.4)
Yes	13 (8.2)	164 (37.6)	20 (60.6)
NA	49 (30.8)	82 (18.8)	1 (3)
**Weight loss, *n* (%)**			
No	79 (49.7)	146 (33.5)	5 (15.2)
Yes	32 (20.1)	216 (49.5)	28 (84.8)
NA	48 (30.2)	74 (17)	0 (0)

BMI, body mass index; NA, not available; SD, standard deviation.

**Figure 1:**
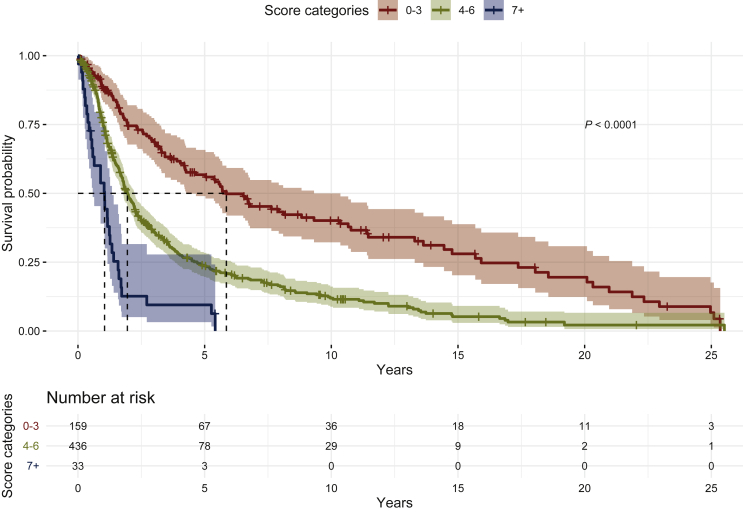
**Kaplan–Meier survival curves by categorized score: low (0-3), medium (4-6) and high (7+) risk**.

**Figure 2:**
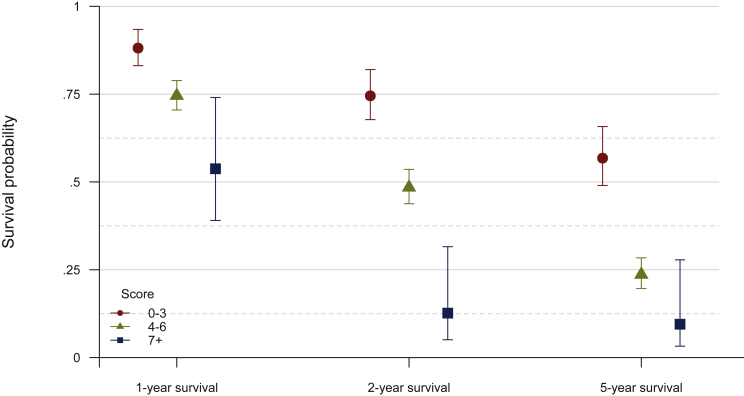
**Survival probabilities for 1-, 2- and 5-year survival by categorized score**.

As seen in Figure 1, the differences in survival probabilities of low-, medium- and high-risk groups were statistically significant (*P* < 0.0001). The survival probabilities for 1 year, 2 years and 5 years according to the risk group are shown in Figure 2. The 1-year survival probabilities for low-, medium- and high-risk groups were 0.88 (95% CI 0.83-0.93), 0.75 (95% CI 0.70-0.79) and 0.54 (95% CI 0.39-0.74), respectively, whereas the 2-year survival probabilities were 0.75 (95% CI 0.68-0.82), 0.48 (95% CI 0.44-0.54) and 0.13 (95% CI 0.05-0.32), and the 5-year survival probabilities were 0.57 (95% CI 0.49-0.66), 0.24 (95% CI 0.20-0.28) and 0.09 (95% CI 0.03-0.28), respectively.

Seven factors (sex, age, BMI, location of primary tumour, stage, stenosis in endoscopy, weight loss) have proven to be statistically relevant in the final Cox proportional hazards regression model with the following parameter estimates: sex: female ♌ = 0.33; location: stomach ♌ = 0.31; stage 3 ♌ = 0.77; stenosis in endoscopy ♌ = 0.38; weight loss ♌ = 0.24; BMI low ♌ = 0.75; age per decade ♌ = 0.12. Cross-validation showed stable results with similar Harrell’s concordance statistics in the validation sets as in the training sets. For ease of use the model coefficients were translated into a simple score (Table 2). Points were allocated to risk factors proportional to effect size. Age categories were selected so that the magnitude of the grouped age effect would at least reach the magnitude of the effects of other risk factors as well as by clinical relevance. In the presence of a risk factor the corresponding value is added to a patient’s total. Absence of a risk factor or unavailability of data add 0 to the total. The total score ranges from 0 to 10.

**Table 2:** VIOLA score

Risk factor	Score
Female sex	+1
Age, years	
30-59	+1
60+	+2
BMI low	+2
Location: stomach	+1
Stage 3	+2
Stenosis in endoscopy	+1
Weight loss	+1

BMI, body mass index; VIOLA, Viennese risk prediction score for Oesophagogastric Localized Adenocarcinoma.

## Discussion

The VIOLA score presents a novel prognostic tool to assist physicians as well as their patients in treatment decisions and, thereby, improve patient management (Table 2). The advantage of the VIOLA score compared with other recently developed prognostic assessments is its clinical feasibility and, thus, its potential to serve as an additional stratification parameter in everyday clinical routine as well as in clinical trials.^22^, ^23^, ^24^, ^25^ To counteract possible bias due to low patient number in some score groups, the cohort could be divided into three risk groups, which further improves clinical feasibility in everyday routine.

Compared with other already established prognostic tools, the VIOLA score is favourable for several reasons. Major contributors to low feasibility in everyday practice of other scores are (i) the need for information that is not available at initial diagnosis, such as specific treatment information^22^^,^^23^^,^^26^^,^^27^; (ii) the need for information that needs to be evaluated solely to calculate the score^28^, ^29^, ^30^; (iii) complicated calculations to get appropriate score results.^31^ Another important issue of existing scores is the fact that known key prognostic factors such as age and sex are often not included and, thus, represent major limitations.^32^ Analyses of the Surveillance, Epidemiology, and End Results (SEER) database showed that older age may decrease survival chances by lower life expectancy in general as well as higher comorbidity burden and even lack of social and connubial support.^33^

Furthermore, all previously established scores were based on limited patient numbers and additional patient heterogeneity concerning stage and routinely assessed histological subtype, which leads to poorer representability.^22^ The large European cohort of the VIOLA score is well in line with current literature^8^^,^^9^^,^^16^^,^^34^ and emphasizes the importance that specific patient and tumour characteristics should be considered when developing prognostic tools.^35^

Interestingly, weight loss as well as changes in BMI were included in the score, which indicates that not only underweight but also the weight loss itself (as well as when the BMI stays within or above the normal limit) is an important risk factor.^15^^,^^34^ It is noteworthy that laboratory parameters, although in part associated with prognosis in univariate analyses, were not included in the final score model. Although the association of laboratory parameters such as tumour or inflammatory markers with the OS has often described to be highly promising, our results emphasize the variability of their prognostic effect in different patient groups.^24^^,^^36^^,^^37^

In addition, common alarm symptoms, although of low predictive value concerning diagnosis,^38^, ^39^, ^40^ play an important role in prognosis of gastroesophageal cancer due to possible reduction of quality of life as well as the nutritional status.^12^

In the era of personalized medicine, specific patient needs and quality of life are in the spotlight of high-end patient care as well as clinical trials. The wide range of survival probability in our homogenous cohort and presence of patients with dismal OS expectance as seen in metastatic settings emphasizes that routinely assessed histological parameters and staging alone are not sufficient for the assessment of OS prognosis.

### Strengths, limitations and future perspective

The rationale and feasibility of this project were based on the recently published Viennese risk prediction score for Advanced Gastroesophageal carcinoma based on Alarm Symptoms (VAGAS) score, which is a risk prediction score for patients with metastatic gastroesophageal cancer.^41^ As patients with advanced disease often have more severe clinical presentation, restricted organ functions depending on metastatic sites as well as different treatment strategies, the VAGAS score is not feasible for patients with regionally limited disease.

Our highly representative large European study cohort presents a major strength of this single-centre analysis, as results might be transferable to other Western cohorts. A further strength of this study is the large time frame in which it was conducted, as this allows for long follow-up periods in a curative patient population. Limitations due to the change of treatment regimen as well as improved patient management throughout the past three decades was minimized as all patients were treated according to the individual decision of an interdisciplinary tumour board. There was no statistically significant difference in the OS throughout the observed period (Supplementary Table S1, available at https://doi.org/10.1016/j.esmoop.2022.100519). Although surgery was associated with the OS in univariate analysis, different treatment approaches including surgery had no statistically significant impact on the OS in the cross-validation model, neither as baseline nor as time-updated variables. In addition, no comparison of different treatment regimen is feasible within the scope of this analysis as a result of individual decision making by the interdisciplinary tumour board.

The major limitations to consider are the retrospective character and missing data, which were minimized by the obligatory detailed documentation of the patient history at the General Hospital Vienna. Yet knowledge about the exact mechanism behind risk factors could not be explored in this analysis. Thus results of this retrospective single-centre analysis have to be verified in a prospective cohort to optimize data collection.

Furthermore, the investigation of longitudinal effects of the prognostic factors included in the score should be addressed in further studies as our results only represent data at the time of first diagnosis.

### Conclusion

As gastroesophageal adenocarcinoma is associated with poor prognosis even in resectable stages, we evaluated the prognostic impact of alarm symptoms, patient and tumour characteristics as well as routine laboratory parameters at first diagnosis in a large European cohort. To improve patient management, we developed a feasible prognostic score, which includes the factors sex, age, BMI, location of the tumour, clinical stage, stenosis in endoscopy and weight loss. The risk prediction score may assess the 1-, 2- and 5-year survival probability and help clinicians as well as patients with treatment and supportive management decisions. More profound knowledge about the patient’s prognosis may endorse more thoroughly follow-up care as well as early supportive treatment arrangements such as psychological and dietary care. Thus the VIOLA score may provide a feasible prognostic tool for everyday clinical routine as well as clinical studies.

## References

[1] Kamangar F., Nasrollahzadeh D., Safiri S. (2020). The global, regional, and national burden of oesophageal cancer and its attributable risk factors in 195 countries and territories, 1990-2017: a systematic analysis for the Global Burden of Disease Study 2017. Lancet Gastroenterol Hepatol.

[2] Wong M.C.S., Huang J., Chan P.S.F. (2021). Global incidence and mortality of gastric cancer, 1980-2018. JAMA Netw Open.

[3] Xie Y., Shi L., He X., Luo Y. (2021). Gastrointestinal cancers in China, the USA, and Europe. Gastroenterol Rep.

[4] Arnold M., Ferlay J., van Berge Henegouwen M.I., Soerjomataram I. (2020). Global burden of oesophageal and gastric cancer by histology and subsite in 2018. Gut.

[5] Gill S., Shah A., Le N., Cook E.F., Yoshida E.M. (2003). Asian ethnicity–related differences in gastric cancer presentation and outcome among patients treated at a Canadian Cancer Center. J Clin Oncol.

[6] Jin H., Pinheiro P.S., Callahan K.E., Altekruse S.F. (2017). Examining the gastric cancer survival gap between Asians and Whites in the United States. Gastric Cancer.

[7] Sung H., Ferlay J., Siegel R.L. (2021). Global cancer statistics 2020: GLOBOCAN estimates of incidence and mortality worldwide for 36 cancers in 185 countries. CA Cancer J Clin.

[8] National Cancer Insititut - Surveillance Epidemiology and End Results Program (2021). Cancer Stat Facts: Esophageal Cancer. https://seer.cancer.gov/statfacts/html/esoph.html.

[9] National Cancer Insititut - Surveillance Epidemiology and End Results Program (2021). Cancer Stat Facts: Stomach Cancer. https://seer.cancer.gov/statfacts/html/stomach.html.

[10] Mokadem I., Dijksterhuis W.P.M., van Putten M. (2019). Recurrence after preoperative chemotherapy and surgery for gastric adenocarcinoma: a multicenter study. Gastric Cancer.

[11] Lou F., Sima C.S., Adusumilli P.S. (2013). Esophageal cancer recurrence patterns and implications for surveillance. J Thorac Oncol.

[12] Maconi G., Manes G., Porro G.-B. (2008). Role of symptoms in diagnosis and outcome of gastric cancer. World J Gastroenterol.

[13] Lordick F., Mariette C., Haustermans K., Obermannová R., Arnold D. (2016). Oesophageal cancer: ESMO Clinical Practice Guidelines for diagnosis, treatment and follow-up. Ann Oncol.

[14] Smyth E.C., Verheij M., Allum W., Cunningham D., Cervantes A., Arnold D. (2016). Gastric cancer: ESMO Clinical Practice Guidelines for diagnosis, treatment and follow-up. Ann Oncol.

[15] Stephens M.R., Lewis W.G., White S. (2005). Prognostic significance of alarm symptoms in patients with gastric cancer. Br J Surg.

[16] Yang D., Hendifar A., Lenz C. (2011). Survival of metastatic gastric cancer: significance of age, sex and race/ethnicity. J Gastrointes Oncol.

[17] Feng F., Tian Y., Xu G. (2017). Diagnostic and prognostic value of CEA, CA19–9, AFP and CA125 for early gastric cancer. BMC Cancer.

[18] Yu Q., Yu X.F., Zhang S.D., Wang H.H., Wang H.Y., Teng L.S. (2013). Prognostic role of C-reactive protein in gastric cancer: a meta-analysis. Asian Pac J Cancer Prev.

[19] Lin J.-X., Lin J.-P., Xie J.-W. (2020). Complete blood count-based inflammatory score (CBCS) is a novel prognostic marker for gastric cancer patients after curative resection. BMC Cancer.

[20] Ouyang X., Dang Y., Zhang F., Huang Q. (2018). Low serum albumin correlates with poor survival in gastric cancer patients. Clin Lab.

[21] Sun H., He B., Nie Z. (2017). A nomogram based on serum bilirubin and albumin levels predicts survival in gastric cancer patients. Oncotarget.

[22] Kologlu M., Kama N.A., Reis E., Doganay M., Atli M., Dolapci M. (2000). A prognostic score for gastric cancer. Am J Surg.

[23] Qian J., Qian Y., Wang J. (2016). A clinical prognostic scoring system for resectable gastric cancer to predict survival and benefit from paclitaxel- or oxaliplatin-based adjuvant chemotherapy. Drug Des Devel Ther.

[24] Jomrich G., Paireder M., Gleiss A., Kristo I., Harpain L., Schoppmann S.F. (2017). Comparison of inflammation-based prognostic scores in a cohort of patients with resectable esophageal cancer. Gastroenterol Res Pract.

[25] Deans D.A.C., Wigmore S.J., de Beaux A.C., Paterson-Brown S., Garden O.J., Fearon K.C.H. (2007). Clinical prognostic scoring system to aid decision-making in gastro-oesophageal cancer. Br J Surg.

[26] Sato S., Oshima Y., Matsumoto Y. (2021). The new prognostic score for unresectable or recurrent gastric cancer treated with nivolumab: a multi-institutional cohort study. Ann Gastroenterol Surg.

[27] Xi M., Liao Z., Deng W. (2017). A prognostic scoring model for the utility of induction chemotherapy prior to neoadjuvant chemoradiotherapy in esophageal cancer. J Thorac Oncol.

[28] Wang P., Wang Y., Hang B., Zou X., Mao J.-H. (2016). A novel gene expression-based prognostic scoring system to predict survival in gastric cancer. Oncotarget.

[29] Li W., Chen Y., Sun X. (2019). Protein expression profiles and clinicopathologic characteristics associate with gastric cancer survival. Biol Res.

[30] Kim K.-M., Lee J., Park S.H., Heo Y.J., Jang J.R., Kim S. (2018). Reproduction of gastric cancer prognostic score by real-time quantitative polymerase chain reaction assay in an independent cohort. Precis Future Med.

[31] del Arco C.D., Muñoz M.E., Roldán E.M. (2021). Proposal for a clinicopathological prognostic score for resected gastric cancer patients. Saudi J Gastroenterol.

[32] Gupta V., Coburn N., Kidane B. (2018). Survival prediction tools for esophageal and gastroesophageal junction cancer: a systematic review. J Thorac Cardiovasc Surg.

[33] Li X., Wang W., Ruan C. (2017). Age-specific impact on the survival of gastric cancer patients with distant metastasis: an analysis of SEER database. Oncotarget.

[34] Okada E., Ukawa S., Nakamura K. (2017). Demographic and lifestyle factors and survival among patients with esophageal and gastric cancer: the biobank Japan project. J Epidemiol.

[35] Costa M.L.V., Ribeiro KdCB., Machado M.A.C., Costa A.C.L.V., Montagnini A.L. (2006). Prognostic score in gastric cancer: the importance of a conjoint analysis of clinical, pathologic, and therapeutic factors. Ann Surg Oncol.

[36] Pan Q.-X., Su Z.-J., Zhang J.-H., Wang C.-R., Ke S.-Y. (2015). A comparison of the prognostic value of preoperative inflammation-based scores and TNM stage in patients with gastric cancer. Onco Targets Ther.

[37] Hirahara N., Matsubara T., Fujii Y. (2020). Comparison of the prognostic value of immunoinflammation-based biomarkers in patients with gastric cancer. Oncotarget.

[38] Rasmussen S., Haastrup P.F., Balasubramaniam K., Christensen R.D., Søndergaard J., Jarbøl D.E. (2018). Predictive values of upper gastrointestinal cancer alarm symptoms in the general population: a nationwide cohort study. BMC Cancer.

[39] Said E.M., Koulaouzidis A., Nicolaides D., Clarkson D., Saeed A. (2006). Predictive value of alarm symptoms in upper gastrointestinal cancer diagnosis: 1294. J Am College Gastroenterol.

[40] Kapoor N., Bassi A., Sturgess R., Bodger K. (2005). Predictive value of alarm features in a rapid access upper gastrointestinal cancer service. Gut.

[41] Puhr H.C., Pablik E., Berghoff A.S. (2020). Viennese risk prediction score for Advanced Gastroesophageal carcinoma based on Alarm Symptoms (VAGAS score): characterisation of alarm symptoms in advanced gastro-oesophageal cancer and its correlation with outcome. ESMO Open.

