# Supplementary figures and images for: Development of an alarm symptom-based risk prediction score for localized oesophagogastric adenocarcinoma (VIOLA score)
Source: ESMO Open. 2022 Jun 24;7(4):100519. doi: 10.1016/j.esmoop.2022.100519 (PMC9434169; doi:10.1016/j.esmoop.2022.100519)

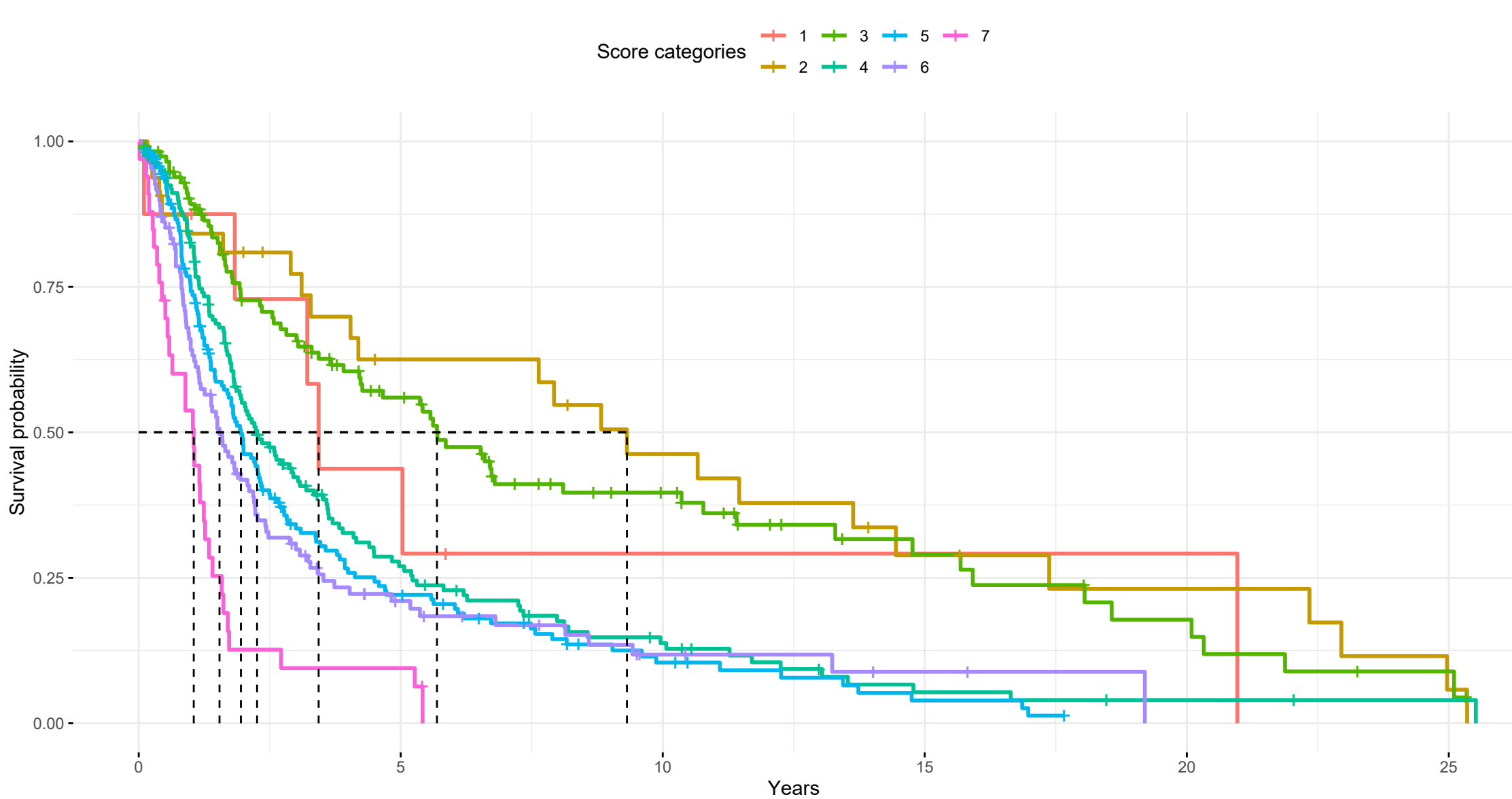

Number at risk

| Score categories | 0   | 5  | 10 | 15 | 20 | 25 |
|------------------|-----|----|----|----|----|----|
| 1                | 8   | 3  | 1  | 1  | 1  | 0  |
| 2                | 32  | 16 | 11 | 6  | 4  | 1  |
| 3                | 119 | 48 | 24 | 11 | 6  | 2  |
| 4                | 163 | 33 | 14 | 4  | 2  | 1  |
| 5                | 165 | 29 | 10 | 3  | 0  | 0  |
| 6                | 108 | 16 | 5  | 2  | 0  | 0  |
| 7                | 33  | 3  | 0  | 0  | 0  | 0  |
|                  | 0   | 5  | 10 | 15 | 20 | 25 |

Years

Supplement: Supplementary Figure S1 [file mmc1.pdf]
