# Supplementary material for: Development of an alarm symptom-based risk prediction score for localized oesophagogastric adenocarcinoma (VIOLA score)
Source: ESMO Open. 2022 Jun 24;7(4):100519. doi: 10.1016/j.esmoop.2022.100519 (PMC9434169; doi:10.1016/j.esmoop.2022.100519)
Supplement: Supplementary Table S1 [file mmc2.docx]

*Supplementary Table 1: Baseline patient and tumour characteristics and their correlation with survival. Abbr: HR = hazard ratio, CI = confidential interval*

| **Characteristic** | **Value** | **p** | **median OS in months/HR (95% CI)** |
| --- | --- | --- | --- |
| **Gender [n (%)]** |  | **0.005** |  |
| male | 446 (71.02%) |  | 30.1 (24.4-35.8) |
| female | 182 (28.98%) |  | 21.9 (17.2-26.6) |
| **Age (years)** |  | **0.002** |  |
| median (SD) | 66 (11.8) |  | 1.013 (1.005-1.021) |
| **Body mass index (BMI)** |  | **0.010** |  |
| within normal limit | 202 (32.2%) |  | 27.2 (20.5-33.9) |
| below normal limit | 18 (2.9%) |  | 14.9 (7.0-22.8) |
| above normal limit | 205 (32.6%) |  | 27.8 (18.8-36.8) |
| **ECOG performance score [n (%)]** |  | **<0.001** |  |
| 0 | 73 (11.6%) |  | 37.3 (28.3-46.3) |
| 1 | 15 (2.4%) |  | 16.9 (16.1-17.7) |
| 2 | 4 (0.6%) |  | 12.9 (0-30.3) |
| n.a. | 536 (85.4%) |  |  |
| **Year of first diagnosis [n (%)]** |  | 0.051 |  |
| 1990-1995 | 59 (9.39%) |  | 55.0 (31.3-78.7) |
| 1996-2000 | 105 (16.72%) |  | 20.6 (15.7-25.5) |
| 2001-2005 | 114 (18.15%) |  | 29.9 (23.0-36.8) |
| 2006-2010 | 147 (23.41%) |  | 23.4 (17.6-29.2) |
| 2011-2015 | 95 (15.13%) |  | 27.4 (20.3-34.5) |
| 2016-2020 | 108 (17.2%) |  | 26.4 (19.1-33.7) |
| **Localization of cancer [n (%)]** |  | 0.673 |  |
| Gastric | 280 (44.59%) |  | 27.2 (21.8-32.6) |
| Gastroesophageal junction | 275 (43.79%) |  | 26.4 (19.5-33.3) |
| Oesophageal | 73 (11.62%) |  | 27.8 (15.1-40.5) |
| **Stage [n (%)]** |  | **<0.001** |  |
| Stage II | 229 (36.46%) |  | 50.5 (36.5-64.5) |
| Stage III | 399 (63.54%) |  | 21.6 (19.4-23.8) |
| **Her-2 status [n (%)]** |  | 0.367 |  |
| positive | 33 (5.25%) |  | 27.5 (17.6-37.4) |
| negative | 140 (22.29%) |  | 40.1 (31.6-48.6) |
| n.a. | 455 (72.45%) |  |  |
| **Systemic therapy [n (%)]** |  | 0.600 |  |
| yes | 361 (57.48%) |  | 25.3 (22.1-28.5) |
| no | 230 (36.62%) |  | 32.5 (23.7-41.3) |
| n.a. | 37 (5.89%) |  |  |
| **Surgery [n (%)]** |  | **<0.001** |  |
| yes | 573 (91.24%) |  | 30.6 (26.1-35.2) |
| no | 54 (8.6%) |  | 11.0 (8.5-13.5) |
| n.a. | 1 (0.16%) |  |  |
| **Radiation therapy [n (%)]** |  | 0.134 |  |
| yes | 80 (12.7%) |  | 23.9 (17.8-30.0) |
| no | 548 (87.3%) |  | 27.8 (23.2-32.4) |
