# Supplementary material for: Development of an alarm symptom-based risk prediction score for localized oesophagogastric adenocarcinoma (VIOLA score)
Source: ESMO Open. 2022 Jun 24;7(4):100519. doi: 10.1016/j.esmoop.2022.100519 (PMC9434169; doi:10.1016/j.esmoop.2022.100519)
Supplement: Supplementary Table S2 [file mmc3.docx]

| **Characteristic** | **Value** | **p** | **median OS in months (95% CI)** |
| --- | --- | --- | --- |
| **Haemoglobin** |  | 0.093 |  |
| within normal limit | 274 (43.63%) |  | 27.8 (20.3-35.3) |
| below normal limit | 120 (19.11%) |  | 20.9 (15.8-26.0) |
| **Platelets** |  | 0.055 |  |
| within normal limit | 310 (49.36%) |  | 27.5 (20.5-34.5) |
| below normal limit | 20 (3.18%) |  | 16.8 (4.9-28.7) |
| above normal limit | 64 (10.19%) |  | 23.3 (16.2-30.4) |
| **White blood cells** |  | 0.138 |  |
| within normal limit | 332 (52.87%) |  | 24.9 (19.4-30.4) |
| below normal limit | 10 (1.59%) |  | 63.2 (0-130.8) |
| above normal limit | 53 (8.44%) |  | 24.3 (14.1-34.5) |
| **C-reactive protein** |  | **0.018** |  |
| within normal limit | 200 (31.85%) |  | 30.1 (20.4-39.8) |
| above normal limit | 165 (26.27%) |  | 22.0 (16.0-28.0) |
| **Albumin** |  | **0.021** |  |
| within normal limit | 300 (47.77%) |  | 27.8 (20.5-35.1) |
| below normal limit | 47 (7.48%) |  | 15.2 (10.8-19.6) |
| **Creatinine** |  | 0.478 |  |
| within normal limit | 345 (54.94%) |  | 26.2 (20.8-31.6) |
| above normal limit | 46 (7.32%) |  | 23.7 (13.1-34.3) |
| **Bilirubin** |  | 0.558 |  |
| within normal limit | 346 (55.1%) |  | 27.5 (21.1-33.9) |
| above normal limit | 26 (4.14%) |  | 21.2 (0-50.4) |
| **Carcinoembryonic antigen** |  | **0.023** |  |
| within normal limit | 211 (33.6%) |  | 27.8 (19.6-36.0) |
| above normal limit | 59 (9.39%) |  | 16.1 (9.205-23.0) |
| **Carbohydrate antigen 19-9** |  | **0.002** |  |
| within normal limit | 169 (26.91%) |  | 33.2 (23.9-42.5) |
| above normal limit | 84 (13.38%) |  | 15.0 (10.9-19.1) |

*Supplementary Table 2: Categorized laboratory parameters and their association with the overall survival (OS).* *Abbr: CI = confidential interval*
