# Supplementary material for: Development of an alarm symptom-based risk prediction score for localized oesophagogastric adenocarcinoma (VIOLA score)
Source: ESMO Open. 2022 Jun 24;7(4):100519. doi: 10.1016/j.esmoop.2022.100519 (PMC9434169; doi:10.1016/j.esmoop.2022.100519)
Supplement: Supplementary Table S3 [file mmc4.docx]

| **Characteristic** | **Value** | **p** | **median OS in months (95% CI)** |
| --- | --- | --- | --- |
| **Dysphagia** |  | **0.018** |  |
| yes | 317 (50.48%) |  | 23.4 (19.7-27.1) |
| no | 202 (32.17%) |  | 29.7 (20.7-38.7) |
| n.a. | 109 (17.36%) |  |  |
| **Weight loss** |  | **0.016** |  |
| yes | 276 (43.95%) |  | 20.8 (17.2-24.4) |
| no | 230 (36.62%) |  | 31.0 (23.1-38.9) |
| n.a. | 122 (19.43%) |  |  |
| **Weakness** |  | 0.798 |  |
| yes | 63 (10.03%) |  | 21.9 (18.0-25.8) |
| no | 457 (72.77%) |  | 26.6 (22.5-30.7) |
| n.a. | 108 (17.2%) |  |  |
| **Gastrointestinal bleeding** |  | 0.915 |  |
| active bleeding | 50 (7.96%) |  | 23.3 (21.1-25.5) |
| ulceration | 71 (11.31%) |  | 26.7 (15.6-37.8) |
| no | 360 (57.32%) |  | 25.3 (20.3-30.3) |
| n.a. | 147 (23.41%) |  |  |
| **Dyspepsia** |  | **0.049** |  |
| yes | 374 (59.55%) |  | 28.2 (22.5-33.9) |
| no | 166 (26.43%) |  | 23.6 (19.4-27.8) |
| n.a. | 88 (14.01%) |  |  |
| **Stenosis in endoscopy** |  | **0.001** |  |
| yes | 197 (31.37%) |  | 21.7 (17.7-25.6) |
| no | 299 (47.61%) |  | 30.9 (22.5-39.3) |
| n.a. | 132 (21.02%) |  |  |

*Supplementary Table 3: Symptoms and their association with the overall survival (OS); Abbr: CI = confidential interval*
