# Supplementary material for: Development of an alarm symptom-based risk prediction score for localized oesophagogastric adenocarcinoma (VIOLA score)
Source: ESMO Open. 2022 Jun 24;7(4):100519. doi: 10.1016/j.esmoop.2022.100519 (PMC9434169; doi:10.1016/j.esmoop.2022.100519)
Supplement: Supplementary Table S4 [file mmc5.docx]

*Supplementary Table 4:* Cross-validation results of stepwise variable selection by AIC. Due to a high level of missingness variables Her2 and ECOG were left out of cross-validation.

|  | **Fold 1** | | **Fold 2** | | **Fold 3** | | **Fold 4** | | **Fold 5** | |
| --- | --- | --- | --- | --- | --- | --- | --- | --- | --- | --- |
|  | β | **p** | β | **p** | β | **p** | β | **p** | β | **p** |
| Gender female vs male | 0.43 | <0.001 | 0.28 | 0.020 | 0.50 | <0.001 | 0.25 | 0.035 | 0.38 | 0.002 |
| Age (per decade) | 0.09 | 0.053 | 0.12 | 0.013 | 0.10 | 0.037 | 0.09 | 0.057 | 0.11 | 0.024 |
| BMI above normal vs normal | 0.00 | 0.994 | -0.05 | 0.681 | 0.04 | 0.774 | -0.12 | 0.371 | 0.04 | 0.787 |
| BMI below normal vs normal | 0.78 | 0.022 | 1.07 | <0.001 | 0.66 | 0.041 | 0.92 | 0.003 | 0.63 | 0.075 |
| Location stomach vs GEJ | 0.40 | 0.003 | 0.29 | 0.024 | 0.21 | 0.100 | 0.35 | 0.004 | 0.31 | 0.015 |
| Location oesophagus vs GEJ | 0.21 | 0.224 | -0.02 | 0.890 | 0.27 | 0.116 | 0.07 | 0.678 | -0.06 | 0.750 |
| Stage 3 vs 2 | 0.71 | <0.001 | 0.64 | <0.001 | 0.71 | <0.001 | 0.80 | <0.001 | 0.84 | <0.001 |
| Surgery |  |  | -0.38 | 0.075 | -0.58 | 0.005 |  |  |  |  |
| Weight loss | 0.25 | 0.052 | 0.22 | 0.070 | 0.20 | 0.101 | 0.17 | 0.157 | 0.37 | 0.003 |
| Weakness | 0.03 | 0.858 |  |  |  |  |  |  |  |  |
| Dyspepsia | -0.17 | 0.189 |  |  | -0.27 | 0.031 |  |  | -0.30 | 0.018 |
| Stenosis in endoscopy | 0.41 | 0.001 | 0.32 | 0.007 | 0.37 | 0.003 | 0.38 | 0.002 | 0.35 | 0.005 |
| Bilirubin above normal | 0.18 | 0.539 |  |  |  |  |  |  | -0.13 | 0.617 |
| Creatinine above normal | 0.05 | 0.813 |  |  |  |  |  |  |  |  |
| Platelets above normal |  |  |  |  |  |  |  |  | -0.28 | 0.121 |
| Platelets below normal |  |  |  |  |  |  |  |  | 0.35 | 0.238 |
| CRP above normal |  |  | 0.29 | 0.038 |  |  | 0.37 | 0.010 | 0.53 | 0.003 |
| CA19-9 above normal |  |  | 0.36 | 0.036 |  |  |  |  |  |  |
|  |  |  |  |  |  |  |  |  |  |  |
|  |  |  |  |  |  |  |  |  |  |  |
|  | **Training** | **Test** | **Training** | **Test** | **Training** | **Test** | **Training** | **Test** | **Training** | **Test** |
| Concordance | 0.646 | 0.627 | 0.641 | 0.663 | 0.640 | 0.602 | 0.651 | 0.606 | 0.669 | 0.577 |
