# Supplementary material for: Development of an alarm symptom-based risk prediction score for localized oesophagogastric adenocarcinoma (VIOLA score)
Source: ESMO Open. 2022 Jun 24;7(4):100519. doi: 10.1016/j.esmoop.2022.100519 (PMC9434169; doi:10.1016/j.esmoop.2022.100519)
Supplement: Supplementary Table S5 [file mmc6.docx]

*Supplementary Table 5:* Final Cox proportional hazards model after cross-validation

|  | **HR** | **95% CI** | **p** |
| --- | --- | --- | --- |
| Gender female vs male | 1.39 | (1.13, 1.71) | 0.002 |
| Age (per decade) | 1.13 | (1.04, 1.23) | 0.004 |
| BMI above normal vs normal | 1.02 | (0.81, 1.28) | 0.871 |
| BMI below normal vs normal | 2.13 | (1.22, 3.71) | 0.008 |
| Location stomach vs GEJ | 1.37 | (1.10, 1.71) | 0.005 |
| Location oesophagus vs GEJ | 1.13 | (0.84, 1.53) | 0.416 |
| Stage 3 vs 2 | 2.15 | (1.73, 2.68) | <0.001 |
| Weight loss | 1.27 | (1.02, 1.57) | 0.029 |
| Stenosis in endoscopy | 1.46 | (1.18, 1.80) | <0.001 |
