# Supplementary material for: Development of an alarm symptom-based risk prediction score for localized oesophagogastric adenocarcinoma (VIOLA score)
Source: ESMO Open. 2022 Jun 24;7(4):100519. doi: 10.1016/j.esmoop.2022.100519 (PMC9434169; doi:10.1016/j.esmoop.2022.100519)
Supplement: Supplementary Table S6 [file mmc7.docx]

*Supplementary Table 6: Median OS and 95% based on score points*

| **Score** | **n** | **Events (death)** | **Median OS** | **95% CI** |
| --- | --- | --- | --- | --- |
| 1 | 8 | 6 | 41.2 | 22.0-n.a. |
| 2 | 32 | 24 | 111.8 | 50.3-208.5 |
| 3 | 119 | 71 | 68.3 | 50.8-124.3 |
| 4 | 163 | 129 | 27.1 | 22.5-36.9 |
| 5 | 165 | 134 | 23.4 | 19.5-28.0 |
| 6 | 108 | 89 | 18.5 | 13.9-26.2 |
| 7 | 33 | 31 | 12.6 | 7.0-16.1 |
