# Supplementary material for: Development of an alarm symptom-based risk prediction score for localized oesophagogastric adenocarcinoma (VIOLA score)
Source: ESMO Open. 2022 Jun 24;7(4):100519. doi: 10.1016/j.esmoop.2022.100519 (PMC9434169; doi:10.1016/j.esmoop.2022.100519)
Supplement: Supplementary Material [file mmc8.docx]

Supplementary Figure 1: Kaplan-Meier survival curves by score points

Supplementary Table 1: Baseline patient and tumour characteristics and their correlation with survival. Abbr: HR = hazard ratio, CI = confidential interval

Supplementary Table 2: Categorized laboratory parameters and their association with the overall survival (OS). Abbr: CI = confidential interval

Supplementary Table 3: Symptoms and their association with the overall survival (OS); Abbr: CI = confidential interval

Supplementary Table 4: Cross-validation results of stepwise variable selection by AIC. Due to a high level of missingness variables Her2 and ECOG were left out of cross-validation

Supplementary Table 5: Final Cox proportional hazards model after cross-validation

Supplementary Table 6: Median OS and 95% based on score points
